# Among the Colleges

**Published:** 1902-01

**Authors:** 


### AMONG THE COLLEGES.

### ONTARIO VETERINARY COLLEGE.

THE Christmas examination of this college was held in the college buildings on Temperance Street on Friday. The following gentlemen, after passing a stringent examination before the usual examining board, were awarded diplomas:

Graduates: Wilson A. Bisbee, Cleveland, Ohio; Alexander Doherty, Ellesmere, Ont.; J. Leonard Faragher, Lorain, Ohio; Albert T. Ford, Neustadt, Ont.; A. P. Lubach, Boonton, N. J.; John L. McCoy, Sussex, N. J.; William D. McMullen, Chilton, Wis.; Arthur E. Melhuish, Toronto; T. C. Neff, Jr., Long Glade, Va.; Robert J. Norton, Owen Sound; J. Arthur Royce, Lincoln, Neb.

---

### BOOK REVIEW.

AN ATLAS OF CLINICAL MEDICINE, SURGERY, AND PATHOLOGY.

JONATHAN HUTCHINSON, F.R.S., General Secretary of the New Sydenham Society, has requested Messrs. P. Blakiston's Son & Co., of Philadelphia, the American agents of the Society, to announce the publication of *An Atlas of Clinical Medicine, Surgery, and Pathology*, selected and arranged with the design to afford, in as complete a manner as possible, aids to diagnosis in all departments of practice. It is proposed to complete the work in five years, in fasciculi form, eight to ten plates issued every three months in connection with the regular publications of the Society. The New Sydenham Society was established in 1858, with the object of publishing essays, monographs, and translations of works which could not be otherwise issued. The list of publications numbers upward of 170 volumes of the greatest scientific value. An effort is now being made to increase the membership, in order to extend its work.

---

Dr. John Wende, of Buffalo, N. Y., was the leader of a November party of thirteen hunters in the Adirondack mountains, where they bagged thirteen deer. Dr. Wende brought down one of the largest bucks with his unerring aim. Future veterinary meetings will be regaled with wonderful stories of deer-stalking.

